# Supplementary material for: Single-Molecule Super-Resolution Imaging of T-Cell Plasma Membrane CD4 Redistribution upon HIV-1 Binding
Source: Viruses. 2021 Jan 19;13(1):142. doi: 10.3390/v13010142 (PMC7835772; doi:10.3390/v13010142)
Supplement: Supplementary file 1 [file viruses-13-00142-s001.pdf]

Supplementary Figures

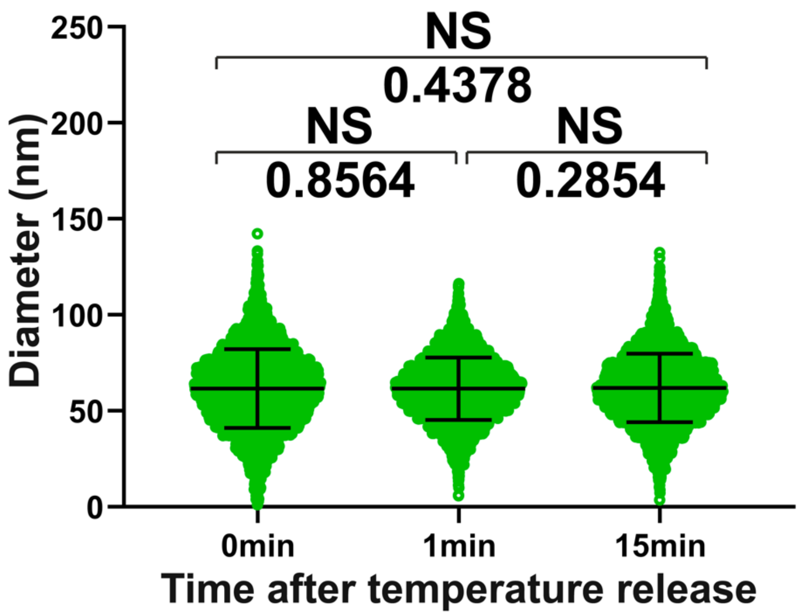

S1. Quantification of CD4 cluster diameters at different time points after releasing the temperature block: Each dot represents 1 cluster. 15 cells were imaged per condition with the mean cluster diameter ± SD indicated by the black bar. A total of 8123, 7822 and 7932 clusters were detected, respectively. The data are representative of at least three independent experiments.

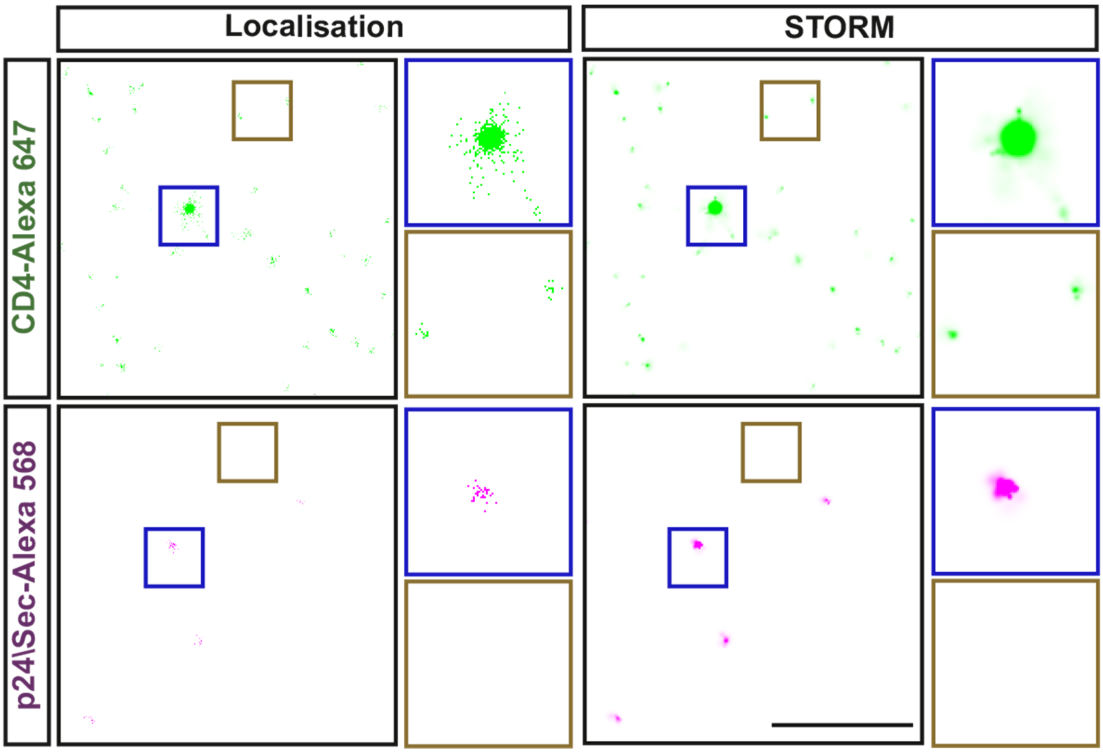

S2. Localisation maps for STORM imaging data. Representative images of: (Left) filtered and drift corrected localisation maps of STORM data, rendered as scatter plots, and (Right) STORM reconstructions of the same localisations, rendered using a 20nm normalised gaussian, of immunolabeled CD4 (top) and p24 (bottom). Scale bars = 1 μm.

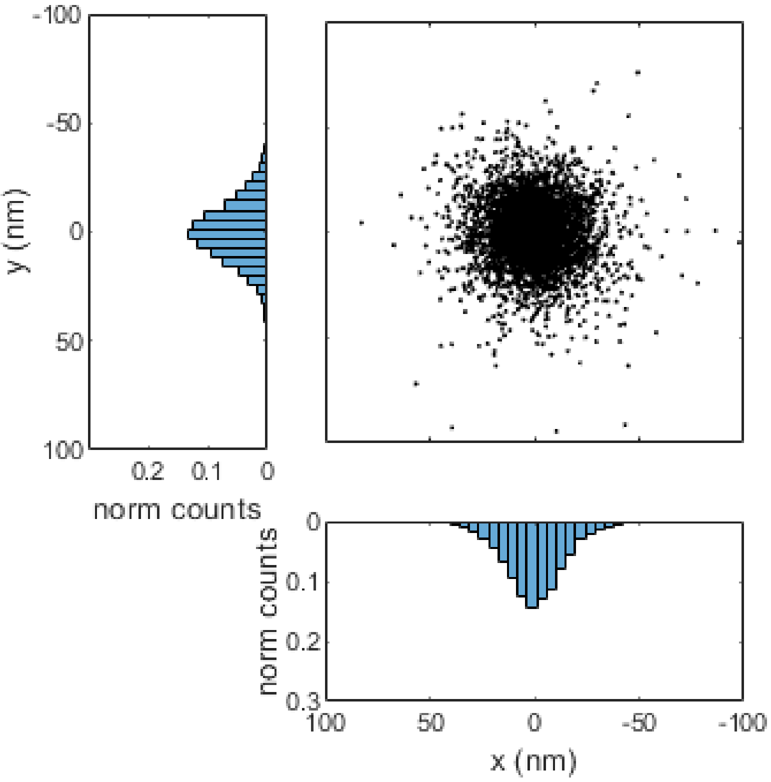

S3. Experimentally obtained localisation precision σ for Alexa Fluor 647. Alignment of localisations from individual Alexa Fluor 647-conjugated antibodies allows an estimation of the average localisation precision with our optical set up and experimental conditions. : $\sigma_{x,y}$ A647 ~ 15 nm.

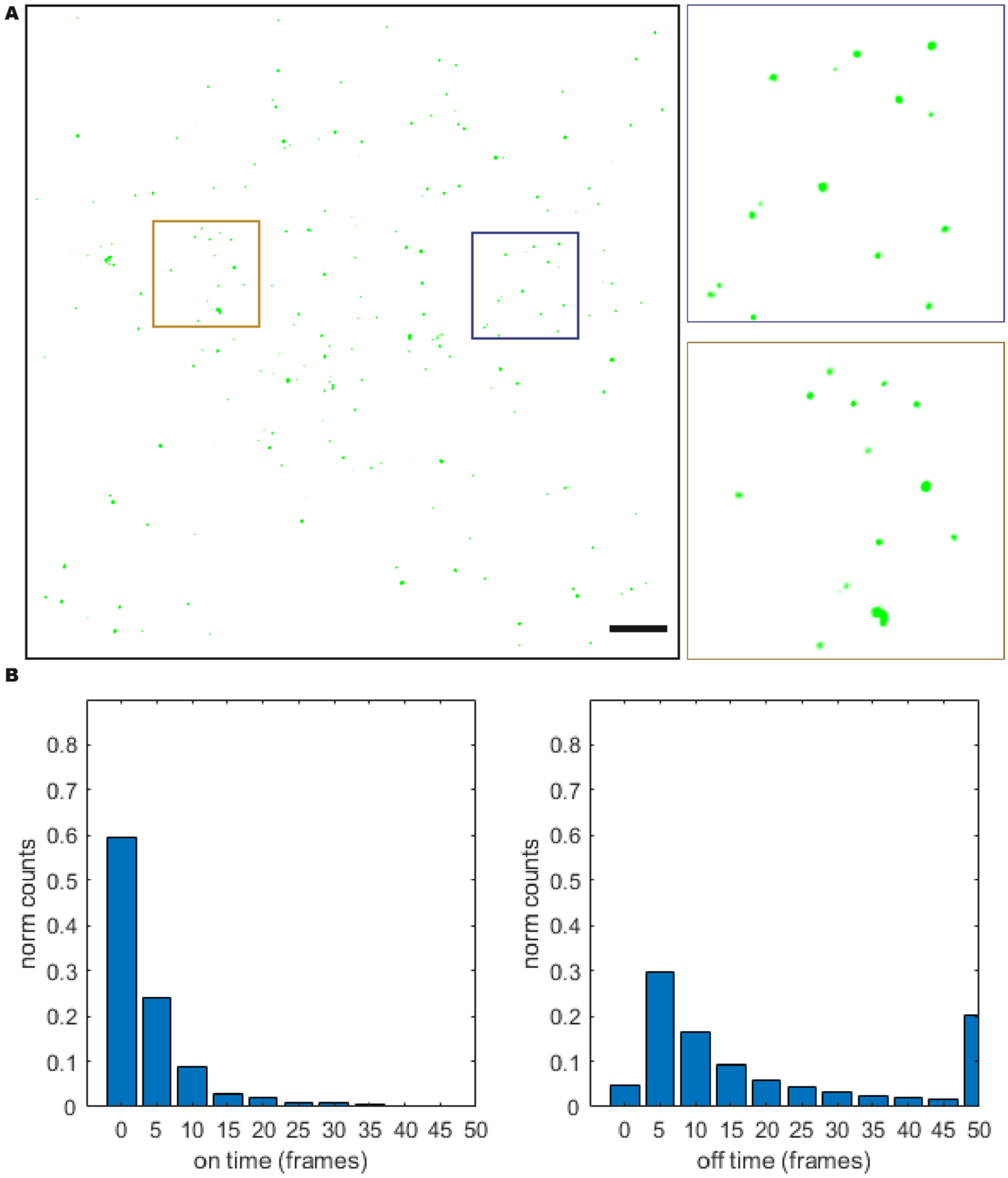

S4. Calibration data for molecule counting from STORM data. (A) Representative STORM image of surface-immobilised AlexaFluor 647-conjugated CD4 antibodies. Distribution of individual molecule on-time (B) and off-time (C) detected. Scale bar: 2 µm.

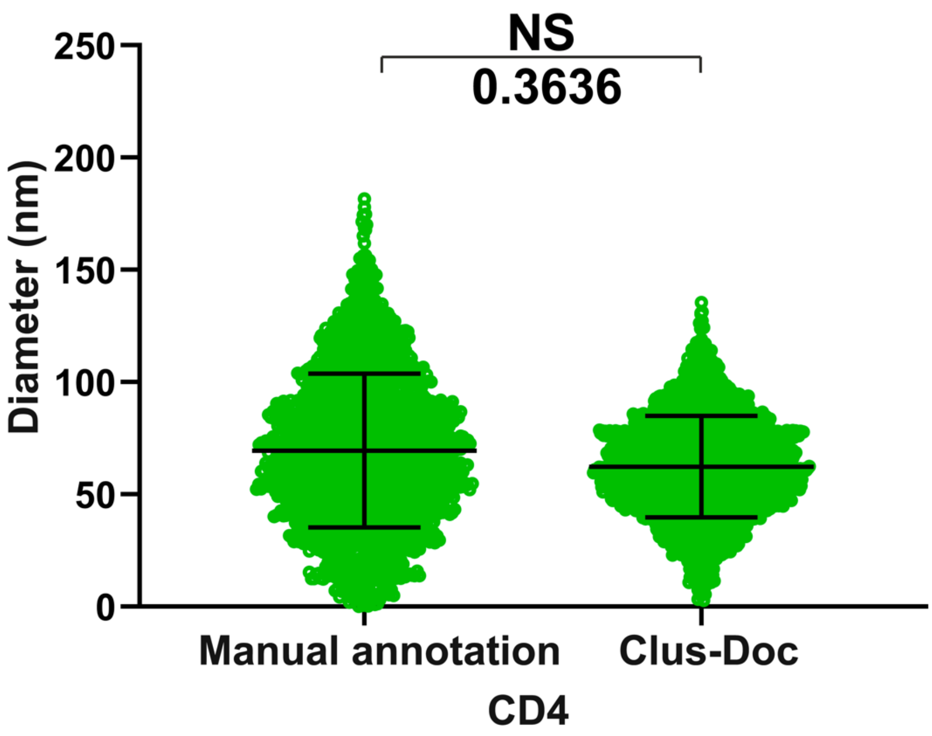

S5. Comparison of CD4 cluster diameters distributions determined by automated Clus-DoC analysis and manual measurements. Each dot represents 1 cluster. 10 cells were measured per condition with the mean cluster diameter ± SD indicated by the black bar. The total number of clusters detected in the manual annotation and DoC were 9731 and 7892, respectively. The data are representative of at least three independent experiments.

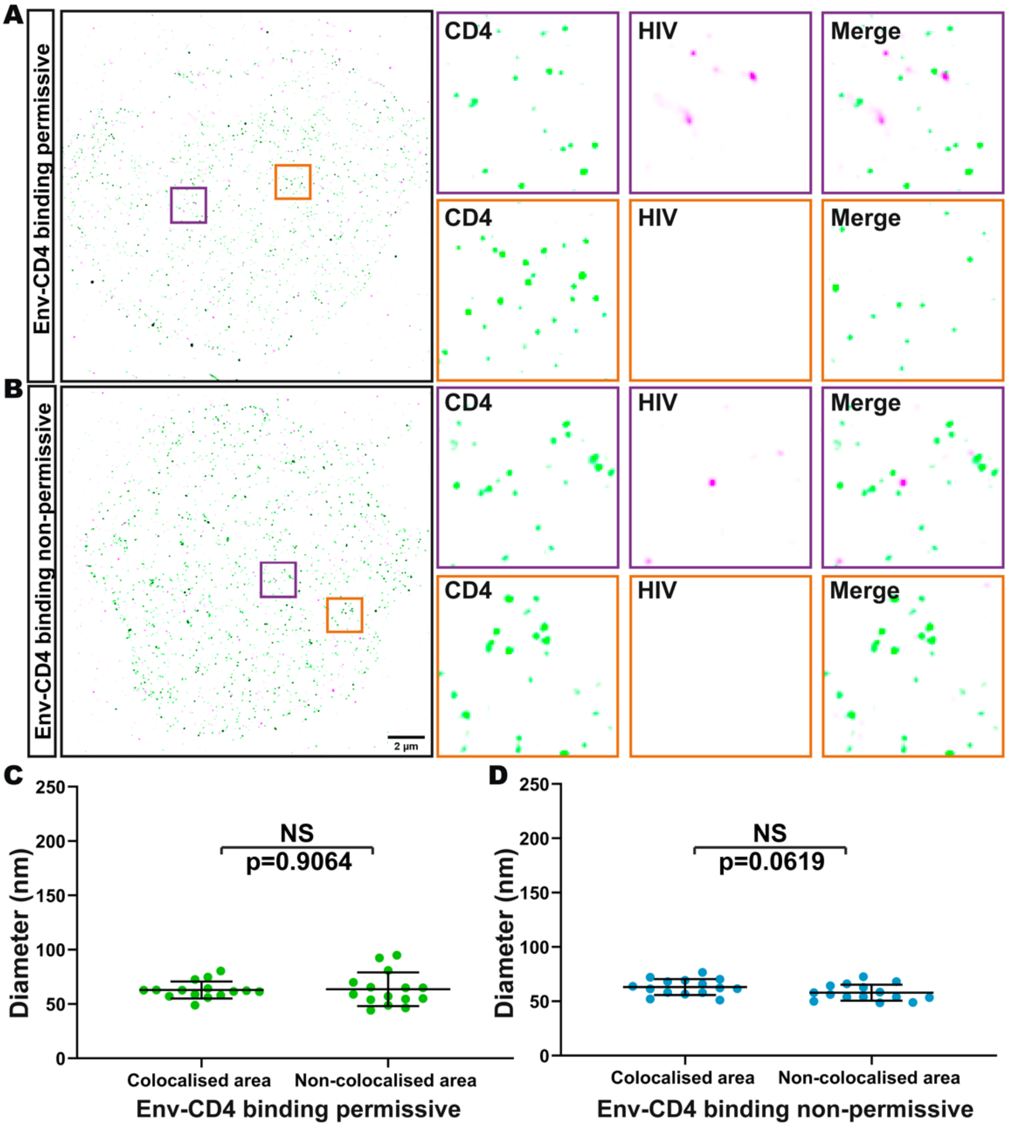

S6. **CD4 does not cluster on HIV-1 treated cells at 4°C. (A,B)** Representative STORM images of CD4 clusters on cells fixed directly without release temperature block. HIV-1 was labelled with anti-p24 and anti-mouse Ig-Alexa Fluor 568. Scale bar = 2 µm. **(C,D)** Quantification of CD4 cluster diameters in the absence (Env-CD4 binding permissive) and presence (Env-CD4 binding non-permissive) of Q4120. In Env-CD4 binding inhibition group, SupT1-R5 cells were pre-incubated with Q4120 (an anti-CD4 antibody that inhibits HIV gp120-CD4 binding). Each dot represents the average diameter of CD4 clusters for one cell. 15 cells were measured per condition with the mean ± SD indicated by the black bar. The data are representative of at least three independent experiments. * $p < 0.05$; ** $p < 0.01$; *** $p < 0.001$; **** $p < 0.0001$.

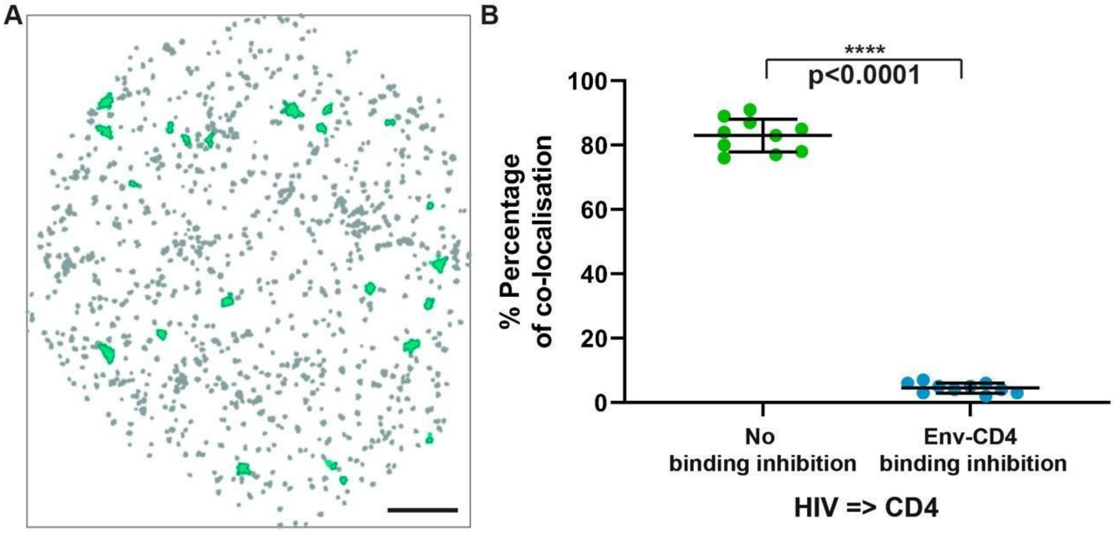

S7. Analysis of CD4 clusters colocalised with HIV. (A) A representative image tracing enlarged CD4 clusters that colocalised with HIV p24 signal on the same cell illustrated in Figure 3B. **(B)** The percentage of HIV particles colocalised to CD4 in the 'Permissive' and 'Non-permissive' as obtained from Clus-DoC co-clustering analysis. Each data point represents a cell. The data are from ten different cells in three separate experiments. * $p< 0.05$; ** $p< 0.01$; *** $p< 0.001$; **** $p< 0.0001$.

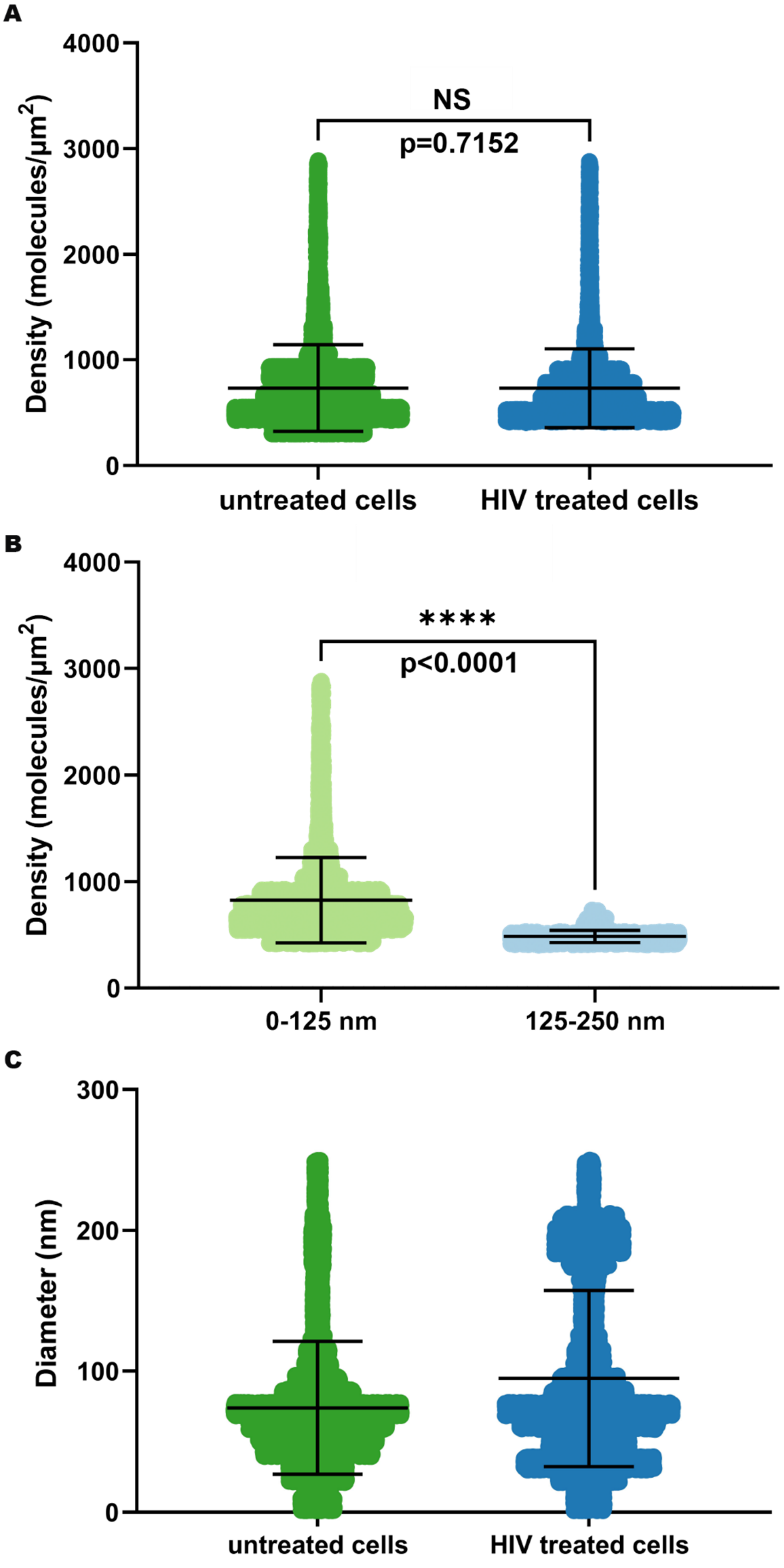
A
4000
3000
2000
1000
0
Density (molecules/µm2)
NS
p=0.7152
untreated cells
HIV treated cells
B
4000
3000
2000
1000
0
Density (molecules/µm2)
****
p<0.0001
0-125 nm
125-250 nm
C
300
200
100
0
Diameter (nm)
untreated cells
HIV treated cells

S8. Diameters and densities of CD4 clusters on HIV-treated cells and untreated cells. (A) Distribution of CD4 molecule density of CD4 molecules on untreated cells and HIV-treated cells. Data from 15 cells in each condition were plotted. **(B)** Distribution of CD4 molecule density for cluster diameter 0-125 nm and 125-250 nm on HIV treated cells. Data from 15 cells. **(C)** Distributions of CD4 cluster diameters on untreated cells and HIV-treated cells. Clusters of ~200 nm were clearly seen in HIV-treated cells but not in untreated cells. Each point represents 1 cluster; Bars represent mean ± SD. * p< 0.05; ** p< 0.01; *** p< 0.001; **** p< 0.0001.

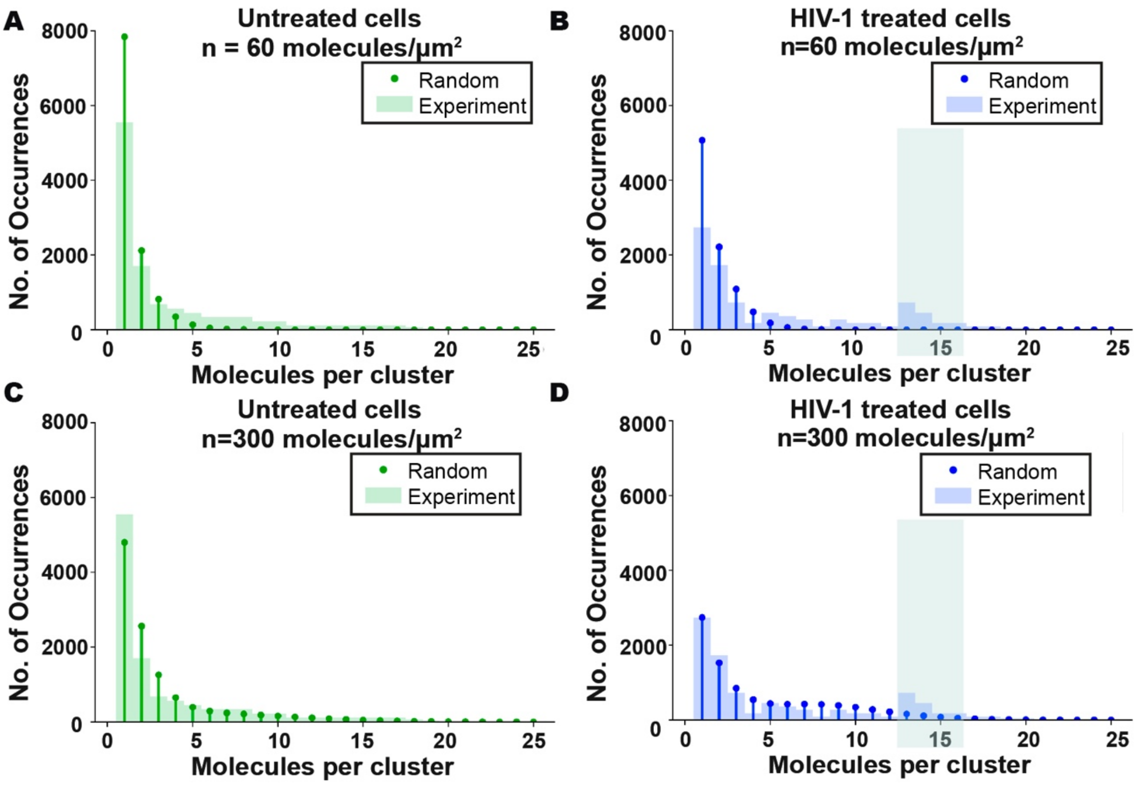

S9. Statistical modelling of CD4 surface distribution. Comparison of expected and measured distributions of numbers of molecules per cluster for untreated cells **(A, C)** and HIV-treated cells **(B, D)**. **(A, B)** represents Poisson distribution data from lower mean density (n=60 molecules/μm²) than showed in Figure 4 (n=200 molecules/μm²). **(C, D)** were Poisson distribution data from higher density (n=300 molecules/μm²) than showed in Figure 4 (n=200 molecules/μm²). The discrepancy between predicted model and experimental data in HIV-treated cells is highlighted in green shading in **B** and **D**. Data from at least three separate experiments and 15 different cells in each condition, respectively.
